# Fecal microbiota transplantation in non-communicable diseases: Recent advances and protocols

**DOI:** 10.3389/fmed.2022.1060581

**Published:** 2022-12-08

**Authors:** Sevag Hamamah, Roxana Gheorghita, Andrei Lobiuc, Ioan-Ovidiu Sirbu, Mihai Covasa

**Affiliations:** ^1^Department of Basic Medical Sciences, College of Osteopathic Medicine, Western University of Health Sciences, Pomona, CA, United States; ^2^Department of Medicine and Biomedical Sciences, College of Medicine and Biological Science, University of Suceava, Suceava, Romania; ^3^Department of Biochemistry, Victor Babeş University of Medicine and Pharmacy Timisoara, Timişoara, Romania; ^4^Center for Complex Network Science, Victor Babeş University of Medicine and Pharmacy Timisoara, Timişoara, Romania

**Keywords:** microbiota transplant, obesity, metabolic disease, inflammatory bowel disease, irritable bowel syndrome, cirrhosis, cancer, FMT protocol

## Abstract

Fecal microbiota transplant (FMT) is a therapeutic method that aims to restore normal gut microbial composition in recipients. Currently, FMT is approved in the USA to treat recurrent and refractory *Clostridioides difficile* infection and has been shown to have great efficacy. As such, significant research has been directed toward understanding the potential role of FMT in other conditions associated with gut microbiota dysbiosis such as obesity, type 2 diabetes mellitus, metabolic syndrome, neuropsychiatric disorders, inflammatory bowel disease, irritable bowel syndrome, decompensated cirrhosis, cancers and graft-versus-host disease. This review examines current updates and efficacy of FMT in treating conditions other than *Clostridioides difficile* infection. Further, protocols for administration of FMT are also discussed including storage of fecal samples in stool banks, inclusion/exclusion criteria for donors, fecal sample preparation and methods of treatment administration. Overall, understanding the mechanisms by which FMT can manipulate gut microbiota to provide therapeutic benefit as well as identifying potential adverse effects is an important step in clarifying its long-term safety and efficacy in treating multiple conditions in the future.

## Introduction

In recent years, there has been an increasing interest in the role of the gut microbiota in health and disease. The term “gut microbiota” refers to all bacteria, archaea, microeukaryotes and viruses that co-exist within the human gastrointestinal (GI) tract (1), while the gut microbiome refers to the collective genomic composition of these microorganisms. Currently, it is estimated that human tract hosts over 100 trillion microorganisms, with a microbiome of approximately 3.3 million unique genes, far surpassing the complexity of the human genome that contains 23,000 genes (2). While initial studies analyzing fetal amniotic fluid suggested no detectable microbial community in the prenatal period (3), recent data provides compelling evidence demonstrating that gut microbial colonization occurs *in utero* (4). After colonization, the gut microbiota develops continuously throughout childhood and adolescence and at the age of 3, it is assumed to closely resemble that of an adult (5). Throughout a individual’s lifetime, the composition of these microorganisms is influenced by a variety of factors including gender, race/ethnicity, location in the GI tract, age and diet. For example, notable differences in gut microbiota species were observed when comparing the microbiota of children who consume healthier, mainly plant carbohydrates, as opposed to children that are adherent to a Westernized diet (6), indicating a heavy influence of lifestyle measures on gut microbiota.

Gut sequencing studies have indicated that richness and diversity of microorganisms in the intestinal tract is closely correlated with human health (7), as colonization of certain bacterial species are shown to be of benefit to the host. Collectively, gut bacteria have been shown to have important roles including, but not limited to, regulating inflammation (8), maintaining gut barrier integrity (9), facilitating digestion, improving insulin sensitivity (10), and enhancing brain health (11). Further, key gut microbiota metabolites, most prominently short-chain fatty acids (SCFA) produced primarily by symbiotic bacterial species, mediate a myriad of these favorable effects on human health (12). The concentration of these SCFA is directly influenced by the relative abundances and deficiencies of certain gut bacterial species. Two main bacterial phyla, Firmicutes and Bacteroidetes, predominate the human gut, accounting for 90% of the species that reside there (13). As such, the Firmicutes/Bacteroidetes ratio, has been often used as a marker to identify correlations with the onset of diseases such as obesity, type 2 diabetes mellitus (T2DM), inflammatory bowel disease and colorectal cancer (14, 15). Imbalances in the intestinal microbiota, also called dysbiosis, play a key role in changes in the Firmicutes/Bacteroidetes ratio, with decreasing microbial diversity, contributing to disease onset. Although numerous studies have shown that the microbiome can recover after certain aggressions, some disturbances may persist leading to negative health outcomes (16). Therefore, significant research has been directed toward understanding the mechanisms by which gut microbiota exert their effects and innovating therapeutic modalities to manipulate these microorganisms in a way that will benefit their host (17, 18).

One such therapeutic modality that has garnered significant interest in the last few decades is fecal microbiota transplant (FMT). FMT aims to restore microbial diversity that is diminished as a result of dysbiosis by delivering fecal microorganisms from a healthy person to a patient. Currently, FMT is primarily indicated in treating recurrent and refractory *Clostridium difficile* infection (CDI) with study findings showing better outcomes than antibiotic treatments (19). Due to its success in treating recurrent CDI, many ongoing studies are investigating the benefits of FMT in non-communicable diseases including metabolic diseases, neuropsychiatric conditions, inflammatory bowel conditions, decompensated cirrhosis, cancers, and graft-versus-host disease (20–24). Collectively, these non-communicable diseases contribute significantly to worldwide morbidity and mortality and often present comorbidly, further worsening patient outcomes and severity of disease (25). Therefore, understanding the safety of, and mechanisms by which, targeted microbiota therapies like FMT restore pathogenic changes can assist in assessing treatment efficacy and help work toward optimizing its’ therapeutic benefits.

Overall, the procedure is deemed to be safe with serious side effects being unusual (26). However, the protocols referring to donor selection methods and the methodology used for fecal transplantation are not consistently or uniformly applied. In many countries, the legislation for using FMT is not well regulated at the national level and most facilities that implement FMT procedures use their own guidelines. The Food and Drug Administration (FDA) and national authority regulations consider stool samples to be drugs and suggest their strict oversight in clinical trials due to risks of accidental pathogen transmission and development of antibiotic resistance (27). Although FMT therapy is constantly simplifying and improving, it remains a complex and expensive procedure, due to the donor selection process, which includes some specific analyses, as well as complex training and administration techniques. Therefore, uniform questionnaires and methodologies to screen donors have been developed to eliminate risks of pathogens and ensure safety prior to transplantation.

In this review, we present the emerging evidence of FMT as a therapeutic modality to improve and restore deleterious effects on gut microbial composition and its resulting effects on the development of pathological conditions beyond recurrent CDI including obesity, diabetes mellitus, metabolic syndrome, neuropsychiatric disorders, inflammatory bowel conditions, cirrhosis, cancers, and graft-versus-host disease. Then, we provide a summary of the guidelines for fecal sample collection and administration involving the donor selection process with inclusion/exclusion criteria, preparation of fecal samples and patient preparation. Lastly, we briefly discuss the risks and benefits of the various methods by which FMT can be administered. Overall, this review highlights recent advances in FMT while providing an outline by which clinicians and scientists can follow when preparing for FMT administration.

## Fecal microbiota transplant and obesity

Over the past several decades, there has been dramatic increases in the prevalence of obesity and its associated metabolic disorders, including type 2 diabetes (T2DM) and metabolic syndrome (28). Cumulatively, these diseases involve significant healthcare costs, with high levels of morbidity and mortality (29). While these diseases are closely associated to human genetics and lifestyle changes, the intestinal microorganisms and their collective genome are now recognized to play an emerging role in their pathogenesis (30). Certain metagenomic sequencing patterns are associated with the phenotype of obesity. In general, health-promoting bacteria like *Lactobacillus, Bifidobacterium, Akkermansia* are reduced, while opportunistic pathogens in the *Enterobacteriaceae, Desulfovibrionaceae*, and *Streptococcaceae* families are elevated (31). These patterns are responsible for changes in the body weight of individuals, suggesting that the modulation of the intestinal microbiome is dynamically correlated with the metabolic phenotype of the human host. Therefore, FMT has been studied as a therapeutic method to replenish beneficial gut microbiota to potentially reverse or prevent further fat accumulation (21). Though it is well-supported that FMT exhibits sustained gut microbial composition changes in obese patients, there is ambiguity in whether the therapeutic modality is actually effective in decreasing body weight (32). In a randomized clinical trial assessing the effects of FMT on adolescents, there was no observed effect of FMT on weight loss at 12 weeks, however, a reduction in abdominal adiposity was detected (33). It should be noted however that *post hoc* analysis of the same patients at 26 weeks with co-existing metabolic syndrome revealed a significant benefit, with 78% resolution of metabolic abnormalities as compared to 23% in the placebo group.

There has also been controversy on whether FMT can induce an obese phenotype by implanting gut microbiota of overweight individuals into lean recipients. In a case study of a patient with CDI undergoing a successful FMT intervention, it was found that the recipient of the stool sample from an overweight donor later developed an obese phenotype (34). Further, FMT studies using twins discordant for obesity, and transfer of microbiota from obese mice significantly increases weight gain and adiposity (35). However, a more recent study evaluating weight gain in patients treated with a single FMT for recurrent CDI found an increased BMI post-FMT. However, the weight gain was not significant, and the increase in BMI was attributed to a return to baseline from the initial weight loss experienced during the active CDI (36). Several studies looked at lifestyle interventions in conjunction with FMT treatment to assess treatment efficacy. For example, dietary and exercise interventions, in addition to FMT in obese patients, results in more advantageous changes in recipient gut microbiota and lipid profile versus FMT alone (20). These improvements were associated with increases in *Lactobacillus* and *Bifidobacterium*, as well as reductions in total cholesterol, as well as low density lipoproteins (LDL). In another study, patients underwent Mediterranean diet-based weight loss programs for 6 months, followed by a weight regain phase from month 6 to 14. Fecal samples were collected during the weight loss period and autologous FMT was performed during the weight gain phase (37). The results showed that autologous FMT with samples obtained during the weight loss period may preserve weight loss and help maintain glycemic control. Still, it is unclear whether most of the benefits observed in this study are a result of dietary and exercise interventions or FMT, though it is likely that lifestyle modifications optimize the therapeutic effects of FMT. Overall, the current literature does not provide clear evidence of the efficacy of FMT in humans as a treatment for reducing BMI directly. It is possible that the length of these studies do not provide enough time for FMT to influence weight changes or that other lifestyle factors are interfering with direct assessment of FMT-related outcomes. However, some studies support the therapeutic role of FMT on metabolic abnormalities and obesity-related sequelae including T2DM and metabolic syndrome, which will be discussed in the next section.

## Fecal microbiota transplant effects on diabetes and metabolic syndrome

There is promising evidence that FMT can exert positive therapeutic effects by attenuating the development and progression of T2DM, T1DM and metabolic syndrome. These metabolic diseases are characterized by a high degree of inflammation, which may eventually lead to insulin resistance and metabolic endotoxemia through damage to the protective intestinal mucosa (38). Induction of a chronic inflammatory state results from an uninterrupted release of cytokines, which damages insulin-sensitive cells in the liver, muscles, and adipose tissue (39). Sequencing studies of gut flora in diabetics has shown particular changes that have been attributed to increase gut permeability and susceptibility to chronic inflammatory states (40). For example, diabetic patients have lower colonies of *Akkermansia muciniphila* compared to healthy controls. *Akkermansia muciniphila* is a Gram-negative bacterium that improves glucose tolerance and insulin resistance. More specifically, *Akkermansia* is found to decrease metabolic endotoxemia by reducing plasma LPS levels and reinforcing the gut barrier, thus exerting its beneficial effects on T2DM (41). Other studies have also shown that the microbiota of T2DM patients show relative deficiencies in *Clostridium, Roseburia*, and *Faecalibacterium prausnitzii*, which are species associated with production of butyrate (42). As such, FMT has been shown to promote the growth of butyrate producing bacteria such as *Roseburia intestinalis* and *Eubacterium hallii*, thus conferring beneficial effect on metabolic diseases (43). Butyrate is a SCFA that is associated with improved insulin sensitivity and attenuates progression of T2DM (42).

It is also important to note that most patients with T2DM take medications to lower blood glucose levels, such as metformin, which have been shown to exert positive effects on gut microbial composition (44, 45). Thus, when FMT is combined with drug administration, the beneficial effect of transplantation from healthy donors to T2DM patients as a direct result from the FMT treatment may be difficult to assess. Most studies assessing the efficacy of FMT are conducted in animal models, with fewer studies in patients with T2DM. For example, a recent study evaluating clinical responses to FMT of 17 human participants, showed that 11 of them (64%) had statistically significant decrease in hemoglobin A1c (HbA1c) and blood glucose, while post-prandial C-peptide, a measure correlated with serum insulin, was elevated (46). Microbiota analysis revealed increases of the genus *Anaerotruncu*s, which has been associated with increased insulin resistance (47). The individuals harboring increased abundance of *Anaerotruncu*s exhibited a better clinical response to FMT intervention (46), indicating that this bacterial genus may be a marker of treatment efficacy in diabetics. Results from another recently conducted study indicated that FMT-induced gut microbiota changes were correlated with improvements in blood glucose in T2DM (48). Importantly, FMT increased the genus *Bifidobacterium* concentrations, shown to have multiple benefits on metabolic health, while reducing *Desulfovibrio* and *Bilophila*, two sulfate-reducing genera associated with increased inflammation and elevated blood glucose.

Similarly, several studies have reported positive effects of FMT in patients with T1DM, which has also been associated with dysbiosis of the gut microbiota (49). For example, in T1DM patients receiving three FMT treatments over the span of 4 months, FMT halted progression of the disease by preventing a decline in residual beta-cell function (50). Specifically, plasma metabolites 1-arachidonoyl-GPC and 1-myristoyl-2-arachidonoyl-GPC were associated with beta-cell preservation, while *Prevotella* was inversely related with beta-cell function. At 12 months post-FMT, stimulated C-peptide serum levels was observed to be at a level similar to the ones measured prior to treatment, indicating the efficacious role of microbiota transplant. In a separate study, Xie et al. reported a case of a 24-year-old patient with T1DM, with severe malnutrition and recurrent abdominal pain, nausea and vomiting, which are symptoms consistent with diabetic ketoacidosis (51). FMT treatment significantly relieved patient’s nausea and vomiting, while also showing gradual improvements in nutritional status and blood glucose control as measured by HbA1c and fasting blood glucose. These clinical improvements were accompanied by drastic improvements in the microbiota composition, that resembled that of the healthy donor. Further, a recent study conducted in a T1DM-induced mice model has shown significant benefits of FMT on male fertility such as improved deficits in spermatogenesis and semen quality (52). This effect was attributed to *Lactobacillus* spp. that were more abundant in the treatment group, leading to increase production of *n*-3 polyunsaturated fatty acid docosahexaenoic acid and eicosapentaenoic acid in the testes, which likely mediate the beneficial effects. Taken together, these findings suggest that FMT administration in patients with T1DM is effective in improving the progression of the disease, its’ metabolic parameters as well as systemic complications that result from disease onset.

In addition to its beneficial effects in improving T1DM and T2DM, FMT has been shown to alleviate symptoms associated with diabetic kidney disease (53). For example, FMT treatment improved multiple parameters including amelioration of insulin resistance, prevention of weight gain as well as reduction of tumor necrosis factor-α (TNF-α) and albuminuria in a mouse model. Intestinal structural integrity was maintained while the abundance of succinate consuming *Odoribacteraceae* bacteria family was increased compared with untreated mice. The succinate consumption capacity of *Odoribacteraceae* is known to cause mitochondrial damage-associated molecular pattern (DAMP), with reductions in the bacterial family being implicated in various inflammatory diseases (54). However, the possible influence of other factors on metabolic outcomes such as lifestyle, pharmacological drugs, in particular metformin, sodium-glucose cotransporter-2 inhibitors, GLP-1 receptor agonists and lipid-lowering drugs, should all be considered when interpreting these results.

**FIGURE 1:**
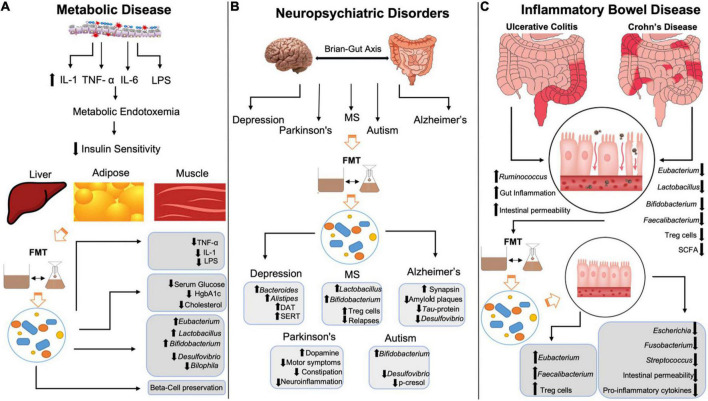
Mechanisms by which FMT restores negative changes in metabolic disease, neuropsychiatric conditions and inflammatory bowel disease. **(A)** LPS-mediated increases in IL-1, TNF-α, and IL-6 leads to metabolic endotoxemia decreasing insulin sensitivity in liver, adipose, and muscle tissue. FMT reduces LPS, IL-1 and TNF-α, lowers serum glucose, HgbA1c and cholesterol levels and preserves beta-cells in T1DM patients. FMT increases *Eubacterium, Lactobacillus*, and *Bifidobacterium* spp., while reducing *Desulfovibrio* and *Bilophila* spp. **(B)** FMT increases dopamine transporter and serotonin transporter expression, while also increasing *Bacteroides* and *Alistipes* in patients in depression. FMT increase *Lactobacillus, Bifidobacterium* and Treg activity in multiple sclerosis patients, while reducing relapses/flares. FMT increases synapsin, amyloid plaques and Tau-protein phosphorylation in Alzheimer’s disease while reducing *Desulfovibrio* spp. FMT increases dopamine while decreasing neuroinflammation, motor and non-motor symptoms in Parkinson’s disease. FMT increases *Bifidobacterium* spp. and reduces *p*-cresol and *Desulfovibrio* spp. in autism spectrum disorder. **(C)** Inflammatory bowel disease is characterized by increased gut inflammation, intestinal permeability and *Ruminococcus* spp. with decreased *Eubacterium, Lactobacillus, Bifidobacterium, Faecalibacterium*, Treg activity and SCFA. FMT restores *Eubacterium* and *Faecalibacterium*, increases Treg activity, while decreasing *Escherichia* spp., *Fusobacterium, Streptococcus*, intestinal permeability, and pro-inflammatory cytokines. IL-1, interleukin-1; TNF-α, tumor necrosis factor α; IL-6, interleukin-6; LPS, lipopolysaccharides; FMT, fecal microbiota transplant; HgbA1c, hemoglobin A1c; MS, multiple sclerosis; DAT, dopamine transporter; SERT, serotonin transporter; Treg, T-regulatory; SCFA, short-chain fatty acids.

In addition to T2DM, FMT has been shown to restore deficits seen in both human and animal model studies of metabolic syndrome (55). For example, in a rat model of fructose-induced metabolic syndrome, FMT reduced metabolic syndrome markers including inflammation and oxidative stress (56). The fructose diet increased *Coprococcus* and *Ruminococcus* levels, both of which were normalized after FMT treatment. *Ruminococcus* is a mucin-degrading species that is associated with pro-inflammatory markers especially when in excess (57). Therefore, some of the anti-inflammatory effects observed in the study may be attributed to reduction in *Ruminococcus* species *via* FMT. In another study that evaluated the effects of FMT on 26 patients with metabolic syndrome, 65% of them showed improved insulin sensitivity 6 weeks after treatment (58), an effect associated with *Bifidobacterium*-induced increases in acetate (59). The specific mechanisms by which FMT exert its benefits on metabolic syndrome are not completely known, however, allogenic microbiota transplant showed improvements of insulin sensitivity *via* methylation of actin-filament associated protein 1 (AFAP1) gene (60), a gene that is associated with altered glucose metabolism. Additionally, FMT recipients with metabolic syndrome showed that treatment helps promote a bacteriophage environment that is similar to that of healthy individuals (61). Taken together, these studies provide strong evidence for FMT in improving insulin sensitivity and glucose metabolism in metabolic disorders (Figure 1).

## Fecal microbiota transplant in neuropsychiatric disorders

Fecal microbiota transplant has been shown to exert a myriad of beneficial effects on psychiatric, neurologic, neurodevelopmental, and neurodegenerative disorders (62) (Figure 1). The bidirectional communication between the brain and the gut, known as the microbiota-gut-brain (MGB) axis is a pivotal component of the neuropsychiatric changes observed after modification of gut microbiota composition. The MGB axis has been shown to influence concentrations of many neuropeptides and neurotransmitters that contribute to altered brain chemistry and disease onset including serotonin (5-HT), dopamine (DA), norepinephrine (NE), epinephrine (Epi) as well as their precursors, receptors, and metabolites (63). Gut microbiota exert effects on the brain neurochemistry *via* neuroactive metabolites such as SCFAs activating vagal afferents, neuroendocrine control of the hypothalamic-pituitary-adrenal axis and pro-inflammatory cytokine mediated inflammation, to name a few (11). As such, FMT has been studied in the setting of neuropsychiatric imbalance to assess the impact of gut microbiota on these pathways and to provide therapeutic benefits to patients.

Mood disorders such as major depressive disorder (MDD), anxiety and bipolar disorders (BD) are multifactorial disorders that are etiologically complex. The lifetime prevalence of generalized anxiety disorder is 33.7% (64), while MDD is 16% and BD is approximately 5% (65). Due to their impact on the global population, significant efforts have been directed toward understanding the role of gut microbiota in the pathogenesis of psychiatric conditions to develop and optimize treatment modalities, including FMT. When evaluating the effects of FMT treatment in mice studies, microbiota from donor stress-induced mice that was transplanted into germ-free mice caused increased anxiety and depression like behaviors and decreased intestinal 5-HT concentrations compared to control animals (66). Both donor stress-induced mice and their microbiota recipients had low levels of *Lactobacillus* and increased *Akkermansia*. *Akkermansia*, when in adequate concentrations, plays an important role in degrading the mucin layer, however, when increased, it can lead to mucin degradation resulting in increased intestinal permeability and susceptibility to endotoxemia (67). Indeed, stress-induced mice have increased neuroinflammation with elevated pro-inflammatory cytokines like interferon-γ (IFN- γ) and tumor necrosis factor-α (TNF-α). Further, the dopamine transporter (DAT) and serotonin transporter (SERT) binding capacities are increased in human subjects with metabolic syndrome undergoing FMT with oral capsules (22). DAT and SERT facilitate reuptake of DA and 5-HT, respectively, to increase neurotransmitter availability in the synaptic cleft. Therefore, the increased bioavailability of these two key neurotransmitters that are heavily implicated in mood disorders, may be a mechanism by which FMT exerts beneficial effects. Additionally, FMT administration to individuals with irritable bowel syndrome (IBS) not only alleviated IBS symptoms but also significantly reduced both depression and anxiety scores (68). Similarly, in the case study reported by Xie et al. (51) and discussed above, the patient with T1DM who underwent FMT treatment also had comorbid depression and treated with duloxetine. Interestingly, during the follow-up post-FMT, the patient no longer experienced depression symptoms. These findings were attributed to alterations in the gut flora that were related to depression, including *Alistipes onderdonkii*, *Bacteroides uniformis*, and *Parabacteroides distasonis.* Further, a recent case report evaluated the effect of FMT in two patients as an adjunctive treatment for depression (69). After 4 weeks post treatment, both patients reported improvement in their MDD symptoms, with one patient reporting benefits up to 8 weeks. Interestingly, the second patient developed a *Bacteroides* enterotype, a species known for its beneficial effects on improving mood *via* production of large quantities of gamma-aminobutyric acid (GABA) (70). Taken together, these findings support the data demonstrating the ability of microbiota transplant to ameliorate symptoms of mood disorders that can be used as a comprehensive treatment to potentially treat multiple comorbidities.

Recent studies have also shown that the intestinal microbiota is involved in the pathogenesis of schizophrenia (71). For example, Zheng et al. (71) have shown that individuals with schizophrenia exhibit reduced microbial diversity and altered microbial composition, notably a decrease of species from the families *Lachnospiraceae* and *Ruminococcaceae.* In the same study, fecal transfer of microbiota obtained from patients with schizophrenia into germ-free mice resulted in increased inhibitory transmitter levels and displayed schizophrenia-like behaviors including increased startle response, locomotor hyperactivity and decreased anxiety and depressive behaviors. These findings are supported by more recent studies showing that healthy mice inoculated with microbiota from patients with schizophrenia developed schizophrenia-like behaviors such as cognitive impairment and psychomotor hyperactivity through increases in the tryptophan degradation pathway, a marker of psychosis onset (72, 73). These changes were accompanied by increased dopamine and 5-HT, in the prefrontal cortex and hippocampus, respectively. Since schizophrenia-like symptoms can be induced through FMT, future studies should be directed toward evaluating the effects of restoring normal gut microbiota in schizophrenic patients *via* microbiota transplant.

Fecal microbiota transplant has also been studied in the context of neurodevelopmental conditions like autism spectrum disorder (ASD), which is characterized by repetitive behaviors with impaired social interactions and communication. Children with ASD have specific plasma and fecal metabolites which are normalized by FMT treatment (74). For example, *p*-cresol sulfate, a fecal metabolite is elevated in children with ASD, an effect that was restored by FMT treatment. *P*-cresol is a harmful microbial metabolite that can cause DNA damage, cell-cycle alterations as well as induce symptoms of autism (75). Recent evidence using a mouse model support the beneficial effects of FMT on reducing *p*-cresol concentrations and rescuing behaviors associated with ASD such as social behavioral deficits and repetitive mannerisms (76). Similarly, FMT performed in 18 children with ASD significantly improved behavioral and GI symptoms up to 8 weeks after treatment (77). This was associated with changes in key bacterial species such as *Bifidobacterium, Prevotella*, and *Desulfovibrio* which persisted for 8 weeks until the end of the study. Importantly, in a follow-up study of the same 18 children, the beneficial effects of FMT in improving behavioral symptoms associated with ASD lasted up to 2 years following treatment (78). Although these trials used small sample size, the findings suggest that FMT is a promising therapy for ASD.

Microbiota transfer trials have also been conducted in the setting of neurologic conditions such as Multiple Sclerosis and Guillain Barre syndrome. For example, transplantation of gut microbiota from intermittent fasting mice, resulted in elevated regulatory T cell (T-reg) activity and increased beneficial species like *Lactobacillus* and *Bifidobacterium*, ameliorated experimental autoimmune encephalomyelitis-induced in MS mice models (79). MS is an demyelinating disease of the CNS that is autoimmune-mediated (80). Therefore, increased T-reg activity after FMT suggests that FMT may modulate the immune system through altering the gut microbial composition. Indeed, a case study of a patient with secondary progressive MS also showed benefits of FMT on disease stability (81). MS is characterized by disease relapses causing flares and disease associated symptoms. This particular patient had recurrent CDI, and seven relapses of MS in the span of 3 years, with worsening neurologic symptoms of balance, bladder function and weakness in extremities. Following FMT treatment *via* rectal enema, the patient did not report any relapses during a 10-year follow-up and had improved functional scores associated with MS severity. Conversely, transplantation of gut microbiota from MS patients into mice induced an MS-like autoimmune disease with less regulatory cytokine production than controls, indicating a critical role of gut microbiota derived influences on MS pathophysiology and its beneficial effects on MS patients (82).

Patients with neurodegenerative disorders such as Alzheimer’s disease (AD) and Parkinson’s disease (PD) may also benefit from microbiota transplant (83). AD is characterized by extracellular aggregation of amyloid plaques and intracellular misfolded tau proteins, which lead to progressive impairments in memory and cognitive decline (84). Recent studies have shown that transfer of fecal microbiota obtained from a rodent model of AD into healthy mice induces symptoms consistent with AD including memory impairment and decreased neurogenesis (85). Gut bacterial dysbiosis and resulting changes in metabolite profile led to an activation of microglia, the macrophages of the CNS. For example, microglia produce Tumor Necrosis Factor-α (TNF-α) and Interleukin-1 (IL-1), to promote neuroinflammation leading to irreversible neuronal damage, a finding characteristic of neurodegenerative disorders (86). In the study described above, transplanting the gut microbes from an AD mouse model potentiated the action of microglia in healthy animals by affecting neurogenesis leading to memory loss. Conversely, transplant of healthy gut microbiota to an AD mouse model decreased Tau-protein phosphorylation and reduced amyloid plaques (87). These effects were associated with significant decrease in key bacterial species from the *Desulfovibrionaceae* family associated with memory loss, as well as increases in other neuroprotective butyrate-producing species. These changes were accompanied by increased synapsin I expression with associated increases in synaptic plasticity and has been found to mitigate mitochondrial damage and memory loss in AD (88). Further, PD that is characterized by dopaminergic neuronal degeneration in the substantia nigra, has been shown to have distinct gut enterotypes, with FMT being proposed as a potential therapeutic modality. For example, in a recent case series of six patients with PD who underwent FMT *via* colonoscopy, it was shown that both motor and non-motor symptom improved in five patients (89). Thus, optimizing gut microbial composition in PD helps to improve dopamine signaling throughout the body, therefore FMT may exert its benefits *via* these pathways (11, 15).

## Fecal microbiota transplant, inflammatory bowel disease, and irritable bowel syndrome

Inflammatory bowel disease (IBD) includes Crohn’s disease and Ulcerative colitis, both of which are characterized by recurrent bouts of intestinal inflammation and their own unique clinical sequelae. Considering the contribution of the gut microbiota to inflammatory states, it is not surprising that certain genera of gut microbiota have been shown to contribute or protect against IBD. For example, pro-inflammatory bacterial species within the *Ruminococcus* genus are elevated in IBD, while SCFA-producing bacterial genera like *Bifidobacterium, Lactobacillus, Eubacterium*, and *Faecalibacterium* are reduced (90). Therefore, targeted microbiota therapy *via* FMT has been studied extensively in the context of IBD (Figure 1).

Ulcerative colitis (UC) is a subset of IBD, characterized by continuous lesions starting from the rectum and extending to the proximal colon (91). Several clinical studies have assessed the efficacy of FMT as a treatment for UC in human subjects. For example, out of 43 patients with UC who received colonoscopic FMT infusion of multidonor samples, 11 patients showed steroid-free clinical remission at 8 weeks, a 19% increase relative to controls (92). Further, FMT *via* colonoscopy administered to 38 individuals with mild to moderate UC resulted in a 23% increase in remission rates at 8-week follow up, with 5 out of 12 patients who achieved remission at 8 weeks exhibited no relapse up to 1 year (24). Though it is important to note that out of 38 patients who received FMT treatment, 3 exhibited serious adverse events including worsening colitis, CDI, resulting in colectomy and pneumonia. A recent study, similarly, evaluating FMT efficacy in 15 UC patients found that, at 8 weeks post FMT, 53% patients in the trial group reported corticosteroid-free remission, compared to only 15% in controls (93). Out of the 10 patients evaluated during a 56-week maintenance phase, four patients continued to have remission by the end of the study. Worsening colitis was again the most common serious adverse effect with two patients developing the condition.

Additionally, microbiota transplant has been recently studied, for the first time, in nine pediatric patients ranging from 4 to 17 years old (94). Out of the nine patients that were treated with FMT and completed the study, eight showed clinical improvement with five patients having clinical remission at 30 weeks, as measured by a Pediatric UC Activity Index score of under 15. However, three patients in the FMT and one patient in the placebo group developed worsening colitis requiring hospitalization and IV methylprednisolone treatment. Since adverse effects have been reported, more recent studies have evaluated both the long-term safety and efficacy of FMT in UC patients. In one prospective pilot study, 10 FMT-treated UC patients were followed over a course of 6–38 months (95). Mayo scores, a marker for UC disease severity, were decreased up to 8 weeks, however this was not statistically significant beyond 6 months. One patient developed Ebstein-Barr Virus within 2 weeks of microbiota transplant, however, no other adverse effects were reported at the time of follow-up, up to 38 months. Important gut microbiota changes after FMT included an increase in the phylum Bacteroidetes, improving the *Firmicutes*/*Bacteroidetes* ratio, with decreases in harmful genera such as *Escherichia.* Long-term efficacy was also assessed by using oral FMT capsules as an adjunctive treatment to FMT *via* colonoscopy (96). The results suggested that using a combination of the two methods of microbiota transplant decreased cytokine production by mucosal associated invariant T (MAIT) cells, up to 36-week follow-up. MAIT cells have been found to be activated in response to active ulcerative colitis, releasing regulatory cytokines such as IL-17 (97). Therefore, reduced MAIT cell activity correlates with the state of remission in ulcerative colitis patients, indicating that FMT may help prevent relapses. Similarly, targeting increased T regulatory cell (Treg) activity is of great interest in IBD (98). Indeed, results from a recent study suggest that FMT introduction of *Faecalibacterium* in UC patients alleviates inflammation by increasing Treg activity, along with decreasing fecal calprotectin, a clinical marker for intestinal inflammation. Taken together, there is a promising body of evidence supporting treatment of UC with FMT in humans, however, further studies need to assess long-term efficacy and safety measures to minimize serious adverse effects before regularly using this therapeutic modality for UC. It is also important to understand and control the factors that predispose disease recurrence in both UC and CD, including anemia, hypoalbuminemia, low peripheral blood lymphocytes and immunosuppression as it may require extra caution with using FMT as a therapeutic intervention (99). Lastly, it should be noted that in UC, FMT *via* colonoscopy appears to be the most effective method as lesions usually begin in the rectum and the distal colon (94).

Crohn’s disease, in contrast to UC, presents with inflammatory lesions that can be present in a discontinuous manner along the entirety of the GI tract, with beneficial outcomes observed in FMT studies that have shown remission in patients up to 24 weeks (100). For example, in 27 patients who received two rounds of FMT one week apart *via* endoscopy and colonoscopy, clinical remission was observed in 18 patients, as measured by serum testing and endoscopy after 8 weeks (101). Importantly, clinically significant difference was observed between the two FMT modalities (endoscopy and colonoscopy). Patients displayed increased microbial richness and diversity, specifically with increases in *Roseburia, Eubacterium*, and *Faecalibacterium*, and reduced *Fusobacterium* and *Streptococcus* after treatment. Interestingly, timing a second FMT intervention in Crohn’s patients who benefited from the first treatment may be of therapeutic value since administration of a second FMT within 4 months of the initial intervention helped maintain clinical benefits (102).

In addition to the intestinal inflammatory conditions, described above, IBS is an unrelated disease, diagnosed clinically and marked primarily by altered bowel habits, either constipation or diarrhea. More recently, IBS has been associated with changes in gut microbiota and microbiota-derived metabolites such as SCFA, bile acids and neurotransmitters like serotonin which is present in abundance within the GI tract (103). SCFA-producing bacterial genus *Bifidobacterium* rich donors have been found to be a key indicator in the response to FMT treatment in IBS patients (104). For example, in a study with 10 IBS patients, six patients achieved a positive clinical response, all of which had donor samples with more *Bifidobacterium.* Similarly, in another fecal transplantation study evaluating 142 IBS patients, the SCFA, butyrate which is inversely correlated with disease symptoms and severity, was found to be significantly increased (105). Therefore, increased SCFA production in recipients after FMT treatment is strongly correlated with treatment efficacy. Other recent studies have assessed the treatment response to FMT in IBS patients. For example, in a study assessing FMT efficacy in 17 patients, 10 were considered responders as measured by the IBS severity index (106). Importantly, in all three of the studies described above no major adverse effects were reported with only some mild self-limiting abdominal, diarrhea or constipation, which are characteristic of IBS at baseline. Further, antibiotic treatment with Ciprofloxacin/Metronidazole or Rifaximin prior to FMT was found to hinder its effects in moderate to severe IBS (107). 15% of patients had improved IBS severity with FMT alone, while the antibiotic treated groups were below 5%. As such, it is important to take the use of antibiotics into account before treating with FMT. Additionally, a recent study has evaluated the efficacy of microbiota transplant in treating IBS with comorbid depression and anxiety (68). A 3-course treatment of FMT *via* oral capsules at 1, 8, and 12 weeks showed improved IBS severity scores and significantly reduced Hamilton anxiety and depression scores at 12-week follow-up, providing more insight into the versatile therapeutic effects of FMT. A summary of the mechanisms by which FMT restores changes in metabolic, neuropsychiatric and inflammatory bowel disease is presented in Figure 1.

## Fecal microbiota transplant, cirrhosis, and hepatic encephalopathy

Cirrhosis develops from long-term liver damage, leading to progressively worsening fibrosis of liver tissue thus preventing normal liver functions. In recent years, significant research has been directed at understanding the microbiota-gut-liver axis, which has been shown to be involved in normal and pathophysiological liver functions (108). Among the proposed mechanisms of microbiota involvement in the onset of cirrhosis is bacterial translocation through intestinal barrier alterations, systemic inflammation, and small intestinal bacterial overgrowth (109). Often, complications of cirrhosis like hepatic encephalopathy and secondary bacterial peritonitis are treated with antibiotics, however, resistance to antibiotic genes is associated with poorer outcomes. Hepatic Encephalopathy (HE) is an indication of decompensated liver cirrhosis that results from excess ammonia buildup leading to altered mental status. Importantly, ammonia producing gut microbes contribute to this process and standard of care includes clearing the ammonia and depleting the culprit bacteria through two medications, lactulose and rifaximin, respectively (110). Theoretically, FMT can introduce beneficial bacteria *via* the gut-liver axis to outcompete ammonia producing microbiota and improve antibiotic resistance. For example, studies have found that FMT can restore antibiotic induced gut microbial dysbiosis (111). In decompensated cirrhosis patients, standard lactulose/rifaximin therapy followed by microbiota transplant with enriched *Lachnospiraceae* and *Ruminococcaceae* resulted in increased SCFA and bile acids with increased microbial richness and diversity. FMT was also found to reduce antibiotic resistance genes, specifically against rifamycin, vancomycin, and beta-lactamases in individuals with decompensated cirrhosis (112). Further, oral capsule FMT was correlated with decreased lipopolysaccharide (LPS) activity and reduced interleukin-6 (IL-6) (113), two inflammatory mediators that can worsen cirrhosis. As such, FMT intervention can improve antibiotic treatment response by lessening the accumulation of resistant bacteria and reduce the overgrowth of harmful bacteria to prevent against LPS-mediated endotoxemia in patients with cirrhosis.

Fecal microbiota transplant has also been studied in patients with recurrent hepatic encephalopathy (HE) as a complication of decompensated cirrhosis. In a study of 10 patients with recurrent HE, cirrhosis severity, cognitive status, liver function and white blood cells were measured in response to FMT without antibiotic pre-treatment compared with the standard of care (SOC) of antibiotic treatment alone (114). FMT donor’s microbiota was enriched with *Ruminococcaceae*, *Bifidobacteriaceae*, and *Lactobacillaceae*, an effect that was observed post-treatment. There was no significant improvement in Model for End Stage Liver Disease (MELD) scores, a measure of cirrhosis severity, however, the SOC worsened MELD scores. FMT treated patients with HE exhibited better cognitive outcomes compared to baseline without significant change compared with SOC group. Importantly, during the 5-month course of the study, no hospitalizations related to altered mental status were observed in the FMT treated individuals, while one was observed in the SOC group. Taken together, these findings suggest that FMT can be an effective treatment in treating cirrhosis and its complications, though more large-scale and longer-term studies are needed.

## Fecal microbiota transplant and cancer

The influence of gut microbiota in tumorigenic pathways has been studied extensively over the years. Several mechanisms by which microbiota can induce carcinogenesis have been put forward, including but not limited to alterations of checkpoint inhibitors, breakdown of gut associated lymphoid tissue and secretion of toxic metabolites (115). For example, intestinal dysbiosis can increase formation of deoxycholic acid, a secondary bile acid with involvement in carcinogenesis *via* increases in tumor cell proliferation and vascular endothelial growth factor receptor expression (116). Conversely, certain gut microbial metabolites have also been shown to ameliorate tumorigenesis. For example, *Bacteroides fragilis* mitigates progression of UC into colorectal cancer through its anti-inflammatory effects (117). This species exerts anti-inflammatory effect by increasing butyrate production and inhibiting activation the NLRP3 inflammasome, a key pro-inflammatory mediator. *Lactobacillus* spp. have also been shown to suppress cell proliferation and inhibit tumorigenesis in a mouse model (118). Therefore, FMT may alleviate the deleterious effects of some factors involved in the progression and development of cancer with a potential role as an adjunct therapy in the future.

Interestingly, two recent studies have found that FMT may improve the response to monoclonal antibody therapy in patients with advanced melanoma (23, 119). Melanoma, in advanced stages, can metastasize and lead to a lack of immune destruction of abnormal cells by T cells after bypassing the programmed cell death-1 (PD-1) immune checkpoint (120). Therefore, targeting the bypassed immune checkpoint inhibitor with anti-programmed cell death protein (Anti-PD1) immunotherapy is effective in long-term treatment, however, anti-PD1 refractory melanoma do exist. In a recent study, combining FMT with anti-PD1 therapy was found to overcome resistance to refractory melanoma (23). Clinical benefits were observed in response to the joint therapy with 6 of 15 patients showing increased CD8 + T cell activation and decreased interleukin-8 myeloid cells, a finding consistent with increased clinical response to anti-PD1 therapy (121). Importantly, gut sequencing studies revealed increased *Bifidobacterium* spp. after FMT treatment, a species associated with synergistic effects on immune checkpoint inhibitors including anti-PD1. Further, a similar study supports these findings by showing that FMT may enhance response to immune checkpoint inhibitor therapy in patients with refractory and metastatic melanoma (119). Study findings show that 3 out of the 10 patients in the clinical trial showed response to the dual therapy with an up-regulation in the immune system activity as measured by T-cell activation, MHCII complex expression and interferon- γ signaling pathways.

Further, chemotherapy treatments are known to cause immunosuppression, leading to infections that require antibiotic therapy. Therefore, in addition to worsening systemic manifestations of cancer, gut microbiota dysbiosis can ensue and FMT may serve as a potential intervention to mitigate complications (122). For example, in 25 patients with acute myeloid leukemia on aggressive antibiotics and chemotherapy, FMT restored microbial richness and diversity, with decreased abundances of pro-inflammatory families *Enterobacteriaceae*, *Enterococcaceae*, and *Veillonellaceae*. No serious adverse events were reported in the study besides mild self-limiting abdominal symptoms indicating treatment safety and its potential adjunctive role in eradicating multi-drug resistance bacteria in cancer patients. Additionally, a case report on a patient with acute lymphocytic leukemia showed similar value on the enhancing effects of gut microbiota in cancer patients who are immunosuppressed (123). In this case, immunosuppressive therapy led to the development of recurrent CDI, which was efficaciously treated with FMT. As such, FMT is a promising therapeutic intervention that may be used in conjunction with cancer immunotherapy to achieve optimal clinical outcomes in refractory cases. Considering that lifetime prevalence of colorectal cancer in long-standing IBD of 30 years is up to 18% (124) and that patients with cirrhosis have a sevenfold increase in risk for developing hepatocellular carcinoma (125), FMT may serve as a preventive measure against carcinogenesis by preventing progression of CD, UC, and cirrhosis.

## Fecal microbiota transplant and graft-versus-host disease

Graft-versus-host disease (GvHD) is an immunologically mediated condition which can result after hematopoietic stem cell transplant (HSCT) when donor bone marrow attacks graft stem cells (126). Interestingly, gut microbiota have been associated with the pathogenesis of GvHD through mechanisms including immune cell and gut microbiota cross-talk across intestinal epithelial cells, stimulation of dendritic cells and Treg cell suppression (127, 128). It is also shown that gut microbiota-derived metabolites such as butyrate and riboflavin are markedly reduced in GvHD (129), with exogenous butyrate administration being shown to attenuate GvHD disease severity by improving intestinal epithelial cells and barrier integrity. Further, MAIT cells, a T-cell subset that is responsive to gut microbiota-derived riboflavin metabolites and present in GvHD target organs, are shown to suppress activity in GvHD through associated decreases in intestinal barrier integrity and IL-17-mediated Th17 expansion (130). More specifically, analysis of colon tissue and stool of MAIT cell-deficient MR1 and IL-17 deficient mice were found to have similar changes in gut microbiota (131). As mentioned earlier, FMT studies on UC patients has been shown to help achieve clinical remission by reducing MAIT cell cytokine production (96), providing a potential role for FMT in GvHD through similar mechanisms. For example, recent longitudinal analysis of FMT performed in a 14-year old GvHD patient showed sustained decreases in *Enterococcus* to undetectable levels over a 3-day period after the FMT (132), while *Faecalibacterium* and *Bacteroides* became more abundant in the patient’s gut. Interestingly, another recent study has shown that *Faecalibacterium* has been associated with high MAIT levels, while *Enterococcus* is correlated with low MAIT levels (133). Overall, these findings suggest that FMT can optimize gut microbial composition to restore MAIT cell function and T-regulatory cell imbalance to exert benefits in GvHD patients.

Due to these findings showing significant involvement of gut microbiota in GvHD, the efficacy and safety of FMT as a therapeutic intervention has been studied. For example, in a study evaluating the effects of FMT on grade IV steroid-refractory GI tract GvHD, the FMT group showed higher rates of clinical remission just 2–3 weeks after treatment and increased the mean survival time to over 432 days as compared to controls (134). These findings were associated with overall increases in the Bacteroidetes/Firmicutes ratio while also increasing other symptoms such as diarrhea and abdominal pain. Of the 23 patients that underwent FMT, two experienced adverse effects including thrombocytopenia and a cardiac event within 7 days of receiving treatment. It is also important to mention that GvHD is a complex pathology and other medications such as immunosuppressants and antibiotics were used concurrently in both the study and control groups, though their effects may vary on an individual basis. Still, the significant improvements in event-free survival as well as overall survival, suggest that FMT administration in GvHD may serve as a viable therapeutic intervention for steroid-refractory GvHD. Another smaller scale study of four patients with steroid resistant acute GvHD reported three complete response and one partial response without adverse events (135). Importantly, changes in gut microbial composition include increases in Faecalibacterium, Bifidobacterium, and Bacteroides, with decreases in Streptococcus, another bacterial species associated with low MAIT cell activity (133). FoxP3 + CD4 + T cells assays showed similar trends in four patients, further supporting the role of effector Treg cells in achieving therapeutic effect in GvHD (135). Further, a larger scale study examining the use of FMT in patients with GvHD, supports the use of microbiota transplant to decolonize antibiotic-resistant bacteria seen in 11 out of 14 patients (136). As such, reduction of the prevalence of antibiotic resistant bacteria may aid treatment of GvHD, should antibiotic treatment be necessary. However, this study does report serious adverse effects though most unrelated to FMT treatment. Septic shock was reported in two patients and Norovirus in another patient, both of which were deemed to be related to FMT, though it should be noted that these patients were severely ill at baseline. Lastly, studies have implemented FMT prior to HSCT to evaluate efficacy in preventing the prevalence and severity of GvHD, however no significant difference in overall survival was found in pre-FMT treatment as compared to controls over a 20-month period (137), indicating that post-HSCT FMT treatment may be more efficacious in clinical outcomes. Overall, there is strong evidence for the use of FMT in controlling the disease severity of GvHD after HSCT.

## Similarities and differences between fecal microbiota transplant studies

Although the studies described in prior subsections evaluate the efficacy of FMT in different non-communicable diseases (Table 1), there are mechanistic similarities in observed benefits as well as trends in gut microbiota profile that correspond to better treatment outcomes. Favorable microbial changes consist of increases in butyrogenic species such as *Faecalibacterium, Eubacterium, Roseburia, Butyrivibrio*, and *Blautia* as well as other beneficial bacteria that produce butyrate precursors like Acetyl-CoA such as *Lactobacillus, Bifidobacterium*, and *Bacteroides* (20, 33, 48, 51, 79, 101, 114, 135, 138). Butyrate strengthens intestinal barrier integrity by inducing AMPK activity to increase tight junction protein expression and improve transepithelial electrical resistance (139, 140). Further, butyrate has been shown to control inflammation by inducing apoptosis of neutrophils, inhibiting mast cell degranulation in the gut and reducing pro-inflammatory cytokines such as IL-6, IL-1 and TNF-α which are elevated in LPS-induced endotoxemia (141, 142). Butyrate also reduces neuroinflammation by upregulating zonulin, occludin, and claudin-5, which are brain tight junction proteins that reduce blood-brain barrier permeability (143). As mentioned, inflammatory states and metabolic endotoxemia contribute significantly to the pathogenesis of metabolic disease, IBD, neuropsychiatric conditions, cancers and GvHD. Therefore, it is not surprising that studies showing therapeutic benefits exerted by FMT share similarities that involve increased butyrogenic species in treatment-responsive individuals with non-communicable diseases. Similarly, the studies discussed in this manuscript show trends in bacterial genera that are reduced in FMT-responsive individuals including *Escherichia, Streptococcus, Desulfovibrio*, and *Bilophila*. Collectively, these species chronically upregulate inflammatory processes through LPS-mediated endotoxemia and reduction of the relative abundances of butyrogenic species, contributing to the development of disease states (144, 145). In addition to trends in gut microbial changes, there are other mechanistic similarities by which FMT may exert its therapeutic effects. For example, four separate studies evaluating the effects of FMT in MS, UC, advanced melanoma and GvHD identify increased MAIT cell activity to the quantity of Treg cells, an important factor in treatment-responsive individuals (23, 79, 96, 134), Further, the incorporation of FMT into the treatment plan of patients with HE and GvHD in adjunction to current regimen can help reduce antibiotic resistance genes to further increase efficacy of standard of care treatments (112, 136). As such, creating targeted changes in gut microbiota to improve gut inflammation and bacterial resistance can help improve treatment-responsiveness to both FMT and concurrent treatment that patients may receive.

**TABLE 1:** Comparison between FMT studies.

Disease studied	Study description	Observed effect	Adverse effects	Gut microbiota alterations	Citation
Obesity	Oral capsule FMT to obese adolescents (*n* = 42) vs. sham treatment (*n* = 45)	No effect on BMI. Reduced abdominal adiposity observed at 12 weeks	Loose stools, abdominal pain, nausea, vomiting, bloody stools	↑*Faecalibacterium prausnitzii, Alistipes, Bacteroides* ↓ *Escherichia coli*	(33)
	Endoscopic FMT on obese patients. FMT (*n* = 20) vs. FMT + lifestyle intervention (LSI) (*n* = 21) vs. sham FMT treatment (*n* = 20)	No significant weight loss in FMT only and sham FMT groups. Reduced liver stiffness, total and LDL cholesterol with weight loss in the FMT + LSI group at 24 weeks	Nausea, vomiting, abdominal pain No FMT related serious adverse effects	FMT alone: ↑ *Faecalibacterium, Roseburia, Eubacterium* FMT + LSI: ↑ *Bifidobacterium, Lactobacillus*	(20)
Type 2 diabetes mellitus (T2DM)	Transendoscopic enteric tube FMT treatment (*n* = 17) on T2DM patients	64% with significant decrease in HgbA1c, blood glucose and uric acid with elevated C-peptide at 12 weeks	none	↑ *Anaerotruncus, Rikenenellaceae*	(46)
	Diet only (*n* = 8) vs. Diet + Oral capsule FMT group (*n* = 8) on T2DM patients	Both groups showed decreased blood glucose and weight loss after 90 days with FMT accelerating the effect	None	↑ *Bifidobacterium, Lactobacillus* ↓ *Desulfovibrio, Bilophila*	(48)
Type 1 diabetes mellitus (T1DM)	Allogenic FMT (*n* = 11) vs. Autologous FMT (*n* = 10) in T1DM patients	Preserved C-peptide levels and beta-cell function at 12 months	None	*Desulfovibrio piger* concentrations predicted beta-cell function	(50)
	Nasojejunal FMT on a 24-year-old patient with T1DM and depression	Improved blood glucose, HgbA1c, constipation, nutritional status Depression symptoms resolved	None	↑ *Bifidobacterium, Blautia, Faecalibacterium, Bacteroides, Eubacterium, Streptococcus* ↓*Alistipes, Escherichia, Parabacteroides*	(51)
Diabetic kidney disease (DKD)	Rectal probe FMT into a mouse model with T2DM and DKD	No weight gain Reduced insulin resistance, TNF-α and albuminuria		↑ *Odoribacteraceae*	(53)
Metabolic syndrome	Oral gavage FMT in metabolic syndrome induced rodent model	Decreased LPS, TNF-α and oxidative stress post-FMT		↓ *Ruminococcus, Coprococcus*	(56)
	Allogenic FMT (*n* = 26) vs. Autologous FMT (*n* = 12) on Metabolic syndrome patients	Improved insulin sensitivity and decreased HgbA1c at 6 weeks post-FMT with no significant difference at 18 weeks	None	↑ *Lactobacillus, Butyrivibrio, Akkermansia* ↓ *Eubacterium ventriosum, Ruminococcus torques*	(58)
Major depressive disorder (MDD)	Oral capsule FMT on MDD patients (*n* = 2)	Both with improved depressive symptoms after 4 weeks and one up to 8 weeks	No serious adverse effects	↑ *Bacteroides, Butyrivibrio, Faecalibacterium* Variable: *Alistipes* spp.	(69)
Autism spectrum disorder (ASD)	Oral or rectal FMT on children with ASD (*n* = 18)	80% with improved GI symptoms Behavioral deficits improved over an 8-week period	Vomiting (*n* = 1)	↑ *Bifidobacterium, Prevotella, Desulfovibrio*	(77)
Multiple sclerosis (MS)	FMT into a mouse model of MS *via* oral gavage	Reduced myelin antigen-specific lymphocytic proliferation, disease severity and spinal cord pathology Increased number of T regulatory cells		↑ *Lactobacillus* spp., *Bifidobacterium pseudolongum, Bacteroides fragilis*	(79)
	Rectal enema FMT in a 61-year-old with secondary progressive MS	Disease stability achieved for 10 years after single FMT Functional composite MS scores improved over 10 years	None	Not assessed	(81)
Alzheimer’s disease (AD)	Intragastric FMT on a mouse model of AD	Reduced Tau-protein phosphorylation and amyloid plaques		↑ Bacteroidetes, *Alloprevotella* ↓ *Akkermansia, Desulfovibrio*	(87)
Parkinson’s disease (PD)	FMT treatment for PD patients (*n* = 6) *via* various delivery methods	Five patients with improvement of motor and non-motor symptoms as early as 4 weeks with significant improvement at 24 weeks	One unspecified adverse event requiring hospitalization	Not assessed	(89)
Ulcerative colitis (UC)	Single FMT *via* colonoscopy and 5 enema FMT per week for 8 weeks (*n* = 42) vs. placebo (*n* = 43) in UC patients	19% increase in remission rates at 8 weeks follow up in the FMT group	Self-limiting GI symptoms in 78% Serious adverse events (*n* = 2)	↑ *Prevotella, Bacteroides* *Barnesiella, Parabacteroides, Clostridium* cluster IV, *Ruminococcus, Blautia* associated with remission *Fusobacterium* and *Sutterella* associated with lack of remission	(92)
	Prepared pooled donor FMT (*n* = 38) vs. autologous FMT (*n* = 35) *via* colonoscopy in UC patients followed by 2 enemas over 7 days	23% increase in steroid-free remission relative to controls at 8 weeks 5/12 patients remained in remission for 1 year	Worsening colitis (*n* = 1) *C. Difficile* infection requiring colectomy (*n* = 1) Pneumonia (*n* = 1)	↑ *Anaerofilum pentosovorans, Bacteroides coprophilus, Alistipes indistinctus, Odoribacter splanchnicus* ↓ *Anaerostipescaccae, Clostridium aldenense*	(24)
	Rectal enema FMT (*n* = 9) vs. placebo (*n* = 6) in pediatric UC patients	Eight patients with clinical improvement measured by the pediatric UC activity index 5 patients with remission at 30 weeks follow up	Development of *C. Difficile* infection (*n* = 2) *Patients already had history of CDI	↑ *Alistipes* spp. ↓ *Escherichia* spp.	(94)
	FMT *via* colonoscopy (*n* = 10) vs. control (*n* = 10) in UC patients	40% improvement in Mayo scores in the FMT treatment group up to 8 weeks but no significant difference to controls at 24 weeks	Ebstein-Barr virus infection	↑ Bacteroidetes, *Prevotella* ↓ Proteobacteria, *Escherichia* spp.	(95)
	Oral capsule FMT after colonoscopic FMT vs. sham oral placebo after colonoscopic FMT	Daily encapsulated therapy extended the durability of FMT-induced changes in gut microbiota Decreased cytokine production by mucosal invariant T cells (MAIT)	Nausea, fever Worsening colitis (*n* = 2)	Similar community-level changes in gut microbiota between donor and recipients	(96)
Crohn’s disease (CD)	Endoscopic FMT followed by colonoscopic FMT one week later in CD patients (*n* = 27)	Clinical remission in 18 patients	No serious adverse effects	↑ *Roseburia, Eubacterium, Faecalibacterium, Bacteroides* ↓ *Fusobacterium, Streptococcus, Clostridium*	(101)
Irritable bowel syndrome (IBS)	FMT *via* colonoscopy in patients with IBS (*n* = 10)	Six patients exhibited improved stool form at 4 weeks Hamilton anxiety and depression scores improved irrespective of IBS response	None	↑*Bifidobacterium* The genus was strongly associated with clinical response to FMT	(104)
	FMT *via* colonoscopy in patients with refractory IBS (*n* = 17)	10 patients showed improved IBS severity index scores of 50 or more points after 12 weeks	Abdominal distention for 2 days after FMT	↑*Akkermansia, Neisseria* ↓ *Desulfovibrio, Delftia*	(106)
	FMT *via* colonoscopy of 30 g samples (*n* = 37) vs. 60 g sample (*n* = 40) in IBS patients already responsive to first FMT	32/37 patients-maintained response to FMT in 1 year 35/40 patients-maintained response to FMT in 1 year	Diverticulitis (*n* = 2)	↑*Eubacterium biforme, Parabacteroides, Bacteroides, Prevotella, Alistipes*	(105)
Hepatic encephalopathy (HE)	Rifaximin/Lactulose followed by rectal enema or oral capsule FMT in cirrhotic patients (*n* = 20)	Increase SCFA and bile acids Reduction in antibiotic resistance genes	Lower HE related complications in FMT group	↑ *Lachnospiraceae, Ruminococcaceae*	(112)
	Single enema FMT in patients with recurrent HE (*n* = 10) vs. Standard of care (SOC) (*n* = 10)	MELD scores remained stable but higher than SOC group FMT treated groups had no HE related hospitalizations while the SOC group had five	No FMT-related adverse effects	↑ *Bifidobacteriaceae Ruminococcaceae, Lactobacillaceae*	(114)
Advanced melanoma	FMT *via* colonoscopy in addition to pembrolizumab in patients with PD-1-refractory-melanoma (*n* = 15)	Six patients showed clinical improvement Increased CD8 + T cell and MAIT cell activation and decreased IL-8 expressing myeloid cells	Hypothyroidism (17.6%)	↑*Bifidobacteriaceae, Ruminococcaceae, Lachnospiraceae* ↓ *Bacteroidaceae, Sutterellaceae*	(23)
	Oral capsule FMT in patients with PD-1-refractory-melanoma (*n* = 10)	Three patients showed clinical response (two partial and one complete)	Mild bloating (*n* = 1)	↑*Enterococcaceae* ↓*Veillonella atypica*	(119)
Acute myeloid leukemia (AML)	FMT treated AML patients (*n* = 25) vs. standard of care (*n* = 20)	FMT is a safe and effective treatment to restore microbiota concentration in AML patients	*Escherichia coli* sepsis (3 months after FMT)	↑*Ruminococceacae, Lachnospiraceae* ↓*Veillonellaceae, Enterococcaceae*	(122)
Graft-versus-host disease (GvHD)	FMT *via* nasojejunal tube to IV steroid refractory GI tract GvHD patients (*n* = 23) vs. controls (*n* = 18)	Higher rates of clinical remission in just 2–3 weeks Increased mean survival to over 432 days compared to controls	Thrombocytopenia (*n* = 1) Cardiac event (*n* = 1)	↑ Bacteroidetes, Firmicutes ↓ Proteobacteria	(134)
	Nasoduodenal tube FMT in GvHD patients (*n* = 4)	Complete response in three patients and partial response in one patient	Paroxysmal atrial fibrillation (*n* = 1)	↑*Faecalibacterium, Bifidobacterium, Bacteroides, Lactobacillus* ↓*Streptococcus*	(135)
	Four FMTs *via* endoscopy in 1 month in a 14-year-old with stage 4 GvHD	Favorable alterations in gut microbiota are present post-FMT in a GvHD patient	None	↑*Faecalibacterium, Bacteroides* ↓ *Enterococcus*	(132)

Though trends of certain bacteria correlating with better disease outcomes were present, these were not consistent in all studies and disease conditions. In T1DM patients, elevation of *Desulfovibrio piger* spp. was correlated with preservation of Beta-cell function (50). Similarly, *Desulfovibrio* was found to be elevated after FMT in children with ASD (77). This is contrary to findings shown in other non-communicable diseases like T2DM, AD, PD, IBS and obesity, that associate elevated *Desulfovibrio* with worse treatment-responsiveness (48, 87, 106). Similarly, variable changes were found in mucin-degrading species, such as *Akkermansia* and *Ruminococcus* (56, 58, 87, 92, 106), as the beneficial effects of these species are concentration dependent (146, 147). Therefore, the post-FMT effects of these bacteria may be specific to both disease and bacterial species, and it is important to consider the relative concentrations to the total microbial diversity within an individual’s gut.

Further, variations in study designs and delivery methods also exist between the studies. For example, some studies evaluate the efficacy of FMT in conjunction with the standard of care or lifestyle interventions (20, 48) while others evaluate the effects of FMT alone particularly in studies evaluating FMT efficacy in metabolic disorders. This makes it difficult to separate the true therapeutic effect of FMT from the effect of lifestyle interventions as gut microbiota are shown to be largely affected by environmental factors, including diet. Also, it is important to note, that due to the severity of some diseases, other treatments were not discontinued during the study, so improvements in patient conditions could involve a combination between FMT and the standard of care (23, 134, 135). Additionally, certain studies used multiple FMT treatments with maintenance therapy (24, 77, 92, 96, 101, 105, 112), while others assess the efficacy of a single FMT treatment (58, 69, 95, 104), with multiple FMTs or maintenance therapies reporting more sustained changes in gut microbiota in the long-term. Preferred delivery methods amongst different diseases were mostly similar, however, varied amongst different diseases. For example, in metabolic diseases, GvHD, CD and depression, FMT was administered through endoscopic approaches or oral capsules (20, 46, 69, 101, 132, 135), while studies evaluating UC and IBS preferred colonoscopy or rectal enema as the distal colon is the most affected (92, 94, 95, 104, 106). The efficacy, advantages and disadvantages of the various delivery methods are further discussed in the following sections.

## Factors for a successful transplant

### Donor selection process

Though FMT is found to be generally effective, it must be performed in a standardized and efficient manner to allow for the provision of safe and correct treatment (148). This is extremely important because patients who need care are often elderly, with comorbidities, which may require urgency in transplantation. Biological sample banks have been developed to facilitate the standardization of the FMT process and ensure the availability and supply of fecal samples on request (149). The existence of these cryogenic biological banks also regulates the availability of willing and healthy donors that meet specific criteria. Although individual donor samples are regularly used in FMT treatment, it has been found that combining fecal samples of multiple donors to create a so called “super donor” augments clinical response to treatment (150). For example, engraftment from both a male and female donor increased microbial diversity, provided more significant enterotype shifts and enhanced metabolic potential of the gut microbial community. More recently, engraftment of the donor microbiota assessed by the strain specific single nucleotide variation in bacterial *rrn* operons has been correlated with improvements in the metabolic health of recipients (151). These methods, however, can be labor intensive and require detailed analysis of fecal samples, which can be performed in a cost-effective manner in organizations with large sample banks and proper equipment. As such, standardized sample banks can optimize and personalize samples from multiple donors to achieve maximal efficacy for patients.

Biological sample banks can be set up directly in individual treatment centers, or they can exist in the form of organizations, such as those in the United States. Until recently, patients who were selected for such treatment usually resorted to fecal samples collected from family members or friends. This approach poses several issues, especially when there is a possibility of donor coercion and ethical and confidentiality concerns regarding the screening of known donors (152). Additionally, family members may carry similar gut microbial profiles as genetic components of certain pathologies and similar environmental factors such as diet and age may yield a similar gut microbial profile to the recipient (153). Though not preferred, FMT may be obtained from related donors, if need be, as there is significant variation in gut microbiota even between family (154). Although the donors with healthy gut microbiota tend to be younger than recipients, age-matching fecal samples can be important, if possible, as variations of microbial composition have been reported in different stages of life (155). Moreover, strict exclusion criteria can be more easily applied to voluntary donors in the community than to those targeted by beneficiaries, as there are more potential candidates without perceived personal obligation between beneficiaries and donors. Further, there is also evidence from safety blood transfusions studies that recipient-directed donors are more likely to be tested positive for infectious disease than unrelated voluntary donors (152), which may also be applied to FMT transmitted infections. It has been found that each stool donation can provide enough fecal samples for up to 8 FMT treatments, thus biological sample banks can be resourceful and maximize donations (156).

**FIGURE 2:**
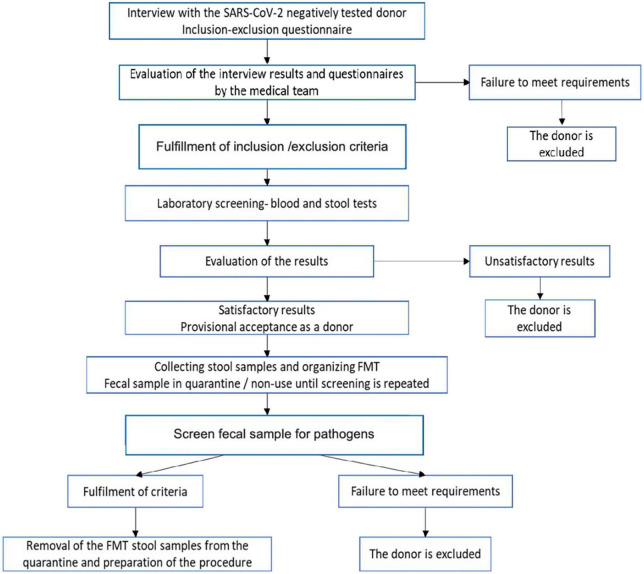
Donor selection and sample preparation flow chart. Patient first undergo screening for SARS-CoV-2, then are assessed with the inclusion-exclusion questionnaire. Donors excluded if the criteria are not met. If inclusion/exclusion criteria met, donors will undergo laboratory blood and stool testing for antibodies/antigens. Donors excluded if they test positive for any antigens/antibodies that can be transmitted through FMT. If blood and stool testing negative, fecal samples will be collected and stored for use in –80°C Celsius freezers. Prior to administration, fecal samples should be checked again for pathogens to ensure safety. If final screening criteria fulfilled, fecal sample is removed from isolation and prepared for the procedure.

Even with the presence of biological sample banks, donor recruitment is an expensive and lengthy process and therefore identifying a target population is recommended to increase donor probability of meeting the inclusion/exclusion criteria (Table 2). This, in itself, presents challenges considering that in 3-year clinical trial only 25% of willing donors, out of 114 candidates assessed in the study were eligible to donate (157). Similarly, in another study, only 12 of 116 (10%) potential donors were eligible to donate fecal samples (158). To maximize the efficiency of the process, the inclusion-exclusion questionnaire is administered followed by the medical examination of the volunteers. The use of a strict protocol for FMT increases cure rate such as seen in the recurrent CDI community-based university hospital study where 86% primary cure rate was observed (159). Therefore, instructions and protocols for fecal sample donation emphasize the importance of extremely rigorous methods for donor selection. Most candidates are excluded after this first screening, thus avoiding the costs of subsequent blood and stool tests. The risk of transmitting an infection through this procedure is minimized by the multi-step screening process. It is also known that several psychiatric, neurological, neurodegenerative, autoimmune, or malignant disorders are associated with certain degrees of dysbiosis and potential donors identified with these disorders should be excluded after screening. To qualify as a donor, potential participants should be interviewed to identify high-risk behaviors and tested for blood and stool samples to exclude any potential infectious agents (Figure 2).

**TABLE 2:** Donor inclusion/exclusion criteria.

*Inclusion criteria*
Age: 18–50 ani (Children under 18 can only donate with parental consent) BMI 18.5–30 kg/m^2^ Should feel good at the time of donation and are similar to age as recipient, if possible

***Exclusion criteria***

**High risk behavior**
○ Use of drugs or other injections without a prescription
○ Exposure to HIV, HBV, or HCV in the last 12 months
○ Unprotected sexual contact or prostitution in the last 12 months
○ Tattoos and piercings made in the last 6 months
○ Incarceration
○ Risk factors for Creutzfeldt-Jakob disease
○ Chronically poor diet ○ Homelessness
○ Pregnancy
○ Frequent activities involving animal (to exclude the risk of transmission of zoonotic infections)
○ Diarrhea (more than three stools per day) among close contacts members (including children) within 4 weeks before donation
○ Person is in a vulnerable group, unable to take care of him/her or unable to protect him/her from significant harm or exploitation
**Current contagious diseases**
○ Fever, vomiting, diarrhea, or other symptoms of infection in the last 4 weeks
○ Vaccinations or injections in the last 8 weeks
○ Blood transfusion, accidental sting with needles exposed to another person’s blood or biological fluids in the last 12 months
○ International travel to countries with poor hygiene, in the last 6 months
**Other conditions**
○ Family members with active gastrointestinal infections
○ Antibiotic treatment in the last 3 months
○ Organ/tissue transplantation ○ *Helicobacter pylori* induced ulcers
○ Gastrointestinal diseases, celiac disease, irritable bowel syndrome, chronic constipation, gastrointestinal tumors, or major gastrointestinal tract surgery
○ Family history of colorectal cancer (more than 2 grade two relatives have/have had the disease)
○ Autoimmune disease
○ Treatment with immunomodulatory drugs ○ Other cancers and active chemotherapy for other diseases ○ History of metabolic syndrome, obesity (BMI > 30 kg/m^2^) or malabsorption ○ Chronic pain syndrome or other neurodegenerative diseases ○ Diabetes ○ Autism ○ Cardiovascular disease, stroke ○ Active or history of mental illness; depression requiring treatment
○ Systemic autoimmunity or atopic diseases
○ Anterior prosthetic implant (e.g., metal heart valve, joint replacement, ventricular-peritoneal shunt, cardiac stent)
○ Allergy to tested antibiotics
○ Known contagious disease or at least 2 weeks after complete recovery from infectious diseases (e.g., chickenpox)

### Inclusion/Exclusion questionnaire

For prospective donors, a physician or nurse will perform a routine medical check-up and evaluate the inclusion/exclusion criteria. Recently, several measures against SARS-CoV-2 have been included as viral particles have been found in the stool of COVID-19 patients and can likely be transmitted (160). As such, prior to any initial assessment or testing, the donor will complete the questionnaire to eliminate the risk of COVID-19 and will be mandatorily tested by RT-PCR or nasopharyngeal exudate to eliminate the risk of SARS-CoV-2 infection. If the potential donor has symptoms associated with COVID-19, it is excluded from the next steps of the donation process until isolation period has passed and RT-PCR negative tests obtained. This criterion extends beyond COVID-19 and any current contagious illness such as those with upper respiratory infections who should not donate fecal samples until they are cleared.

There are several important criteria within the inclusion/exclusion questionnaire. Individuals with history of conditions that have been associated with gut microbial dysbiosis should be excluded This include those discussed in prior subsections like metabolic syndrome, T2DM, neuropsychiatric conditions, IBD, IBS, malnutrition and cancer. Patients with autoimmune diseases and atopic conditions such as asthma and eczema should also be excluded as these conditions have associated changes in gut microbiota and can potentially predispose recipients to new allergic reactions (161). Further, patients on immunomodulatory drugs or chemotherapy are part of the exclusion criteria as the resulting immunosuppression can lead to opportunistic infections that can be transferred to recipients.

High-risk behaviors are another important part of initial screening and should be taken seriously. These behaviors include use of injection drugs, recent tattoos or piercings, incarceration, recent travel to countries with poor hygiene, homelessness, high-risk sex behaviors and those in vulnerable groups (162). Individuals in these categories unfortunately are at higher risk for transmissible infection and should not donate fecal samples. Further, after initial screening, stool and blood testing should be performed to rule out several transmissible conditions. Blood testing evaluates routine labs like complete blood counts, liver function tests, rate of erythrocyte sedimentation, electrolytes, urea and creatine, as well as transmissible diseases such as human immunodeficiency virus, hepatitis, syphilis and human T-cell lymphocytic virus (158). Although these conditions are primarily viral, FMT has been shown to transfer viral communities among donors and recipients and therefore screening prior to treatment is imperative (163). Fecal testing includes screening for *C. difficile* toxin, *cryptosporidium* antigen, a fecal ova/cyst/parasite panel, norovirus immunoassay, rotavirus immunoassay, adenovirus assay and routine bacterial culture for enteric pathogens (158). Stool testing for the presence of antibiotic resistant bacteria, especially those associated with higher mortality rates such as methicillin-resistant *Staphylococcus aureus*, Vancomycin-resistant *Enterococcus*, carbapenamase-producing *Enterobacteriaceae* and extended-spectrum beta-lactamase *Escherichia coli*, should be completed as up to 55% of qualified donors have had multidrug-resistant organisms (164). Failure to screen for these bacterial species have been related to transfer of antibiotic resistance to recipients resulting in bacteremia and even death (165). Importantly, in 2019 more rigorous screening protocols were added for asymptomatic *Helicobacter pylori*, a leading cause of peptic ulcer disease, which was detected in up to 44% *via* nested PCR (164). As such, urea breath test, the gold standard for *Helicobacter pylori* diagnosis, is recommended in stool testing.

## Criteria for obtaining and processing fecal samples

For use for microbial transplantation, feces must be collected correctly and safely. An important step in ensuring the success of a FMT is the quality of the sample delivered to the beneficiary. Therefore, it is important that the procedure for obtaining samples for FMT contains a set of regulations, including access to high quality facilities, with standard operating procedures that allow the safe processing of samples by trained staff.

After the completion of the screening and the identification of the donors, the stool samples are collected from the donor within a maximum of one month from the analysis. It is recommended that, before donation, people involved in this process take a mild laxative to facilitate the elimination of stool the next day (166). Samples will be collected using a specific kit and should be free of water, urine or blood. Donors have the option to donate to the default location for collection or at home; for collection at home, the donor is required to follow an additional set of instructions that involves an important step which is maintaining the sample in a cooled area or with ice packs, and the obligation of returning the stool sample to collection centers, within 1 h after defecation. Subsequently, the stool sample can be stored for up to 8 h at 4°C, without affecting the bacterial flora (167). Studies have shown that fecal samples contain viable bacteria even after 6 months of storage in at least –80°C and, in many cases, cryogenic samples were as effective as freshly harvested ones (168).

**FIGURE 3:**
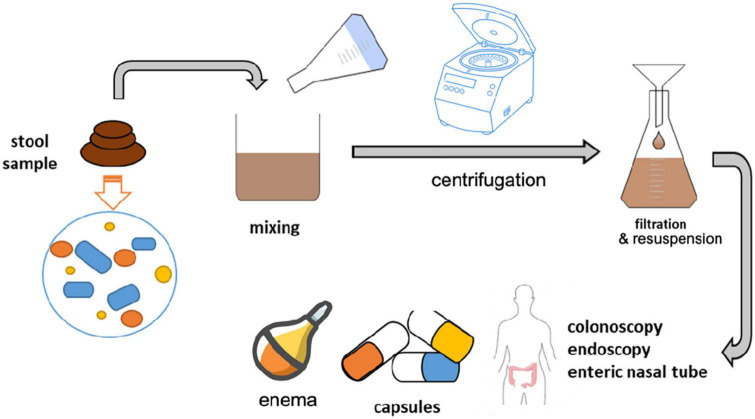
Fecal sample preparation: After testing for contaminants and pathogenic species, stool samples are mixed with saline and glycerol with a final ratio of 25% stool, 65% saline, and 10% glycerol. Mixing is done through a blender or centrifugation to homogenize the sample with the adequate ratio of substrates. The mixture is then filtered and resuspended until it is fully homogenized. The homogenized solution is stored in cryotolerant containers at –80°C for FMT use. When ready for use, the solution is loaded into capsules or delivered through colonoscopy, endoscopy, enteric nasal tube or enema.

Generally, for FMT, a minimum of 50 g stool sample is required for successful transplant, though studies have shown efficacy with 30 g (169). This stool sample is combined with saline and glycerol in a stool, saline, and glycerol ratio of 25, 65, and 10%, respectively (170). The proportion has been well established so that the amount of stool in suspension has a suitable viscosity so that it can be manipulated and transplanted into the colon, using the biopsy tube of the colonoscope (171). In addition to the ability to homogenize the stool sample, glycerin is required to maintain bacterial viability in frozen biological samples (172). The procedure requires homogenizing the fecal sample with saline and glycerin for 1 min, using a rotary blender (Figure 3). The blender mixing process produces a fine suspension that can be loaded into a catheter-syringe and inserted into the patient’s colon through the biopsy channel of the colonoscope (170, 173). If a blender or autoclave is not available, the suspension can be prepared by manually mixing the stool sample, saline and glycerol in a special bag, used only for this purpose. Similarly, the stool can be homogenized directly in the storage bag with a spatula or in a bottle (174). Although these methods are easier, they can result in suspensions with large particles, which will block the syringe at the time of transplantation; therefore, in order to eliminate this risk, it is necessary to filter the suspension. After homogenization, the sample is divided into cryotolerant containers, which is stored at –80°C (175). When storing samples, it is advisable to use larger containers than the amount of homogenized liquid as cryogenic solutions may increase in volume (173). Another preservation method involves filtering and centrifuging the obtained suspension, followed by resuspension of the concentrated formula in saline and 12.5% glycerol for cryoprotection of frozen formulas (170). Medical personnel performing the stool preparation operation for fecal transplantation must wear disposable microbiological protective equipment including masks, gloves, insulating suits, etc. The procedure will be performed in the hood or, if possible, in an anaerobic environment, in order to protect the anaerobic bacteria. Further, continuous, and efficient sanitation of the equipment involved in the fecal sample preparation process is essential to avoid cross-contamination.

A secured document will be completed for each donor, and it will include information about the donor, contact details, screening results, and identification number (173). If the donor is unknown to the patient, the general data protection regulation (GDPR) recommendations for anonymization will be followed. The information kept confidential is necessary to identify the traceability of evidence in the event of the recipient’s illness and to properly record evidence and donors. Containers with stool samples will have the number and date of collection written on the labels. Research has shown that frozen fecal material is shown to be as effective as freshly collected samples, therefore samples should not be refrozen after defrosting.

## Patient preparation for fecal microbiota transplant

The preparation of patients for FMT also involves administration of antibiotics at least 3 days before the procedure (166), with stoppage of antibiotics at least 24–48 h prior to transplantation. Further, it is important to understand the effects of the medications that the patient is on that may affect bowel habits and increase the likelihood of complications from the procedure. In addition to stopping antibiotics, iron-containing supplements and anti-coagulants should be stopped if delivery route presents a risk of bleeding. The delivery routes are discussed in more detail in the following subsection. Preparation is dependent on delivery methods. The FMT administration team will be required to provide the patient with the risks and benefits of the procedure with discussion of possible complications correlated with each specific delivery route. The patient can then provide informed consent and sign the consent form. If FMT is delivered by colonoscopy, the bowel is prepared in advance with polyethylene glycol to improve the visualization of the colon (166). Those undergoing FMT administration *via* flexible sigmoidoscopy may also benefit from a bowel lavage. Further, bowel preparation may be useful to clear out *C. difficile* as well for upper GI administration, however studies have shown that other routes of administration can be effective without it (176). The standard dose is set by each institution or medical team but varies from 50 to 100 g of donated fecal material, diluted in 250–500 ml infused.

## Fecal microbiota transplant delivery methods

Fecal microbiota transplant can be performed using invasive procedures, such as colonoscopy, sigmoidoscopy, endoscopy, or can be administered by retention enema, ingestion of capsules and nasal tubes (Table 3).

**TABLE 3:** Fecal microbiota transplant (FMT) delivery methods with advantages and disadvantages.

Delivery methods	Advantages	Disadvantages
Nasal tube	Delivery without sedation Low costs Useful if patient unable to swallow	Risk of vomiting and aspiration Lowest efficacy
Endoscopy	– It can be performed safely in patients at risk of post-colonoscopy complications	– Discomfort associated with administration Risk of vomiting and aspiration Risk associated with the procedure Requires sedation
Capsules	Non-invasive process Time efficiency Convenient administration High cure rates Can be repeated easily	Risk of vomiting and aspiration Capsules can be large with higher mass
Colonoscopy	Most effective method Can deliver fecal sample to the right colon Most preferred invasive method Can screen for other etiologies simultaneously	Risks for intestinal perforation and bleeding Needs sedation Need for a board-certified gastroenterologist More costly Important to stop anti-coagulants Contraindications Requires bowel preparation
Sigmoidoscopy	No sedation required Can screen for distal 1/3rd colonic pathologies simultaneously	Risk for intestinal perforation and bleeding Inability to use the area on the right side of the colon Need for a board-certified gastroenterologist Important to stop anti-coagulants Bowel preparation recommended
Retention enema	Low costs High tolerability Without sedation Easily repeated Can be done in pediatric patients that cannot have a colonoscopy	– Retention difficulties in some cases Inability to use the area on the right side of the colon

The most effective mechanism of FMT administration is *via* colonoscopy with success rates described to be between 84 and 93%, with a recent meta-analysis reporting a cure rate of 95% (177). Besides bowel preparation, the procedure is almost always performed with sedation and does present low risks for complications including intestinal perforation, bleeding and side effects associated with anesthesia (178). Contraindications to colonoscopy include recent surgeries, recent myocardial infarction, hemodynamic instability, recent bowel injury (179). It is recommended that the fecal sample is deposited in the right colon, if possible. Peristaltic contractions will move the fecal sample along the colon and gut microbiota contained within the sample will be distributed throughout the gut (166). Even with the risks associated with colonoscopy, it is the most preferred invasive method due to the ability to perform colon screening simultaneously (180).

Sigmoidoscopy also allows for deposition of the fecal sample within the colon, however only the left colon can be accessed *via* this delivery route (181). Sigmoidoscopy presents similar risks of complications including intestinal perforation and bleeding, but the procedure can be performed with sedation, so risks associated with anesthesia are not pertinent. Although this method is not used often, a recent case report shows a successful case of FMT treatment *via* sigmoidoscopy on a patient with ischemic colitis secondary to CDI (182). This delivery method may be important if patients have severe right colon disease or obstructions proximal to the hepatic flexure.

Endoscopy provides an invasive method of fecal sample delivery through the upper GI tract into the proximal duodenum (183). The risks associated with endoscopy are similar to those of colonoscopy, with intestinal perforation, bleeding and side effects associated with anesthesia being the most common, although they are rare in general. Further, introduction of samples into sedated patients poses a risk for aspiration as well, therefore patients should be kept upright after the procedure (184). A previous study using endoscopy to infuse fecal samples to recipients showing an 81% cure rate after the first infusion and 94% cure rate after multiple duodenal infusions *via* endoscopy (185). Although repeated infusions showed similar efficacy to colonoscopy, the need for sedation and risks of repeated procedure makes this method less efficacious in comparison.

Administration of fecal samples *via* retention enema is another viable option with high cure rates for recurrent CDI of 87%, though found to be less effective than colonoscopy and capsules (177). Enemas have minimal risks of complications and can be repeated, which increases efficacy. It is important to instruct patients to resist the urge to defecate and retain the enema for as long as possible. Studies have also recommended that retention enemas may be a good adjunctive FMT treatment in addition to upper GI administration (186).

Oral capsules, a highly preferred method by patients, as it is the least invasive with high levels of efficacy of up to 92% (177). Several studies have compared the cure rates of colonoscopy with oral capsules, particularly in treating recurrent CDI (187). Benefits of oral capsules include ease of administration and repeated treatments; however, a single treatment can require up to 30 capsules (188). Capsule shells are resistant to gastric acid; thus, proton pump inhibitors are not required though they can be effective in facilitating treatment (189). Adverse effects are much fewer than invasive methods with rare cases of nausea and vomiting reported (187). Finally, FMT with nasal gastric/duodenal tube is another option, though it has been shown to have the lowest cure rates for CDI at approximately 78% (177). Although this method is not preferred, it can be indicated when patients are unable to swallow oral capsules.

## Challenges, limitations, and future perspective

Fecal microbiota transplant has been proven effective in the treatment of numerous diseases. Initially tested and used for the treatment of recurrent and refractory CDI, the advantages of its use have broadened its applicability, with ongoing clinical trials to assess efficacy in non-communicable diseases including obesity, type 2 diabetes mellitus, metabolic syndrome, neuropsychiatric disorders, inflammatory bowel disease, IBS, decompensated cirrhosis, GvHD and even cancers, as discussed throughout this review. Though generally safe, there are adverse events reported throughout the literature. As such, regulated stool banks have been developed with specific inclusion/exclusion criteria and protocols for sample preparation and FMT administration to limit transfer of unwanted pathogens. Still, there remains a lot that is unknown and missing knowledge gaps that has prevented this therapeutic modality from obtaining FDA approval for treatments beyond CDI.

Although gut sequencing technology is continuously advancing, the gut microbiota comprises a vast amount of species, with a considerable amount of the bacterial species and their role and functions still being unknown (190). This is a significant limitation as it is possible that some of these species have a major impact on the variable outcomes seen between studies. Likewise, significant efforts and progress have been made in identifying key bacterial species that are correlated with better outcomes in FMT. It is clear, however, that the overall response to treatment involves a complex interplay between the gut microbial composition and the host (190). These unknown factors add an extra layer of variability, hence the need for detecting and uncovering the functions of other bacterial species, yet unknown, that may exert a major role in health and disease, whether alone or *via* bacterial competition and host interaction. Further, due to the large variations between donors, stool samples that are heterogeneous, the results may be less reproducible and long-term outcomes may be transient (191). To eliminate variability in fecal samples, recent studies have suggested that synthetic microbiota communities may be the future of FMT (191, 192). By creating a structurally controlled bacterial community, similar samples can be reproduced, pathogenic microorganisms can be eliminated and bacteria that is deemed to be beneficial can be cultured at a larger scale. With continuous technological advances, it is possible that fecal sample preparation may be standardized through synthetic microbial communities to provide a balance of optimal gut microbiota concentrations for recipients.

Overall, the data within the scientific literature for FMT for treatment of a variety of conditions is promising. Though longer-term evaluations exist, most studies have assessed efficacy of treatment for 6 months or less and using small samples. Further, changes in gut microbiota are highly dependent on environmental factors including diet and geographic locations which can enhance efficacy or prevent the desired response to FMT treatment (193). As such, future studies should take these factors into account to define long-term safety of the treatment more clearly and provide lifestyle recommendations that can be used in conjunction with FMT to maximize its therapeutic benefits.

## Author contributions

SH: conceptualization, writing original draft, and editing. RG: conceptualization, resources, writing original draft, and editing. AL: writing – review and editing. I-OS: conceptualization and writing – review. MC: conceptualization, writing – review, and supervision. All authors made a substantial, direct, and intellectual contribution to the work, and approved it for publication.
